# Associations between prenatal mercury exposure and early child development in the ALSPAC study

**DOI:** 10.1016/j.neuro.2016.02.006

**Published:** 2016-03

**Authors:** Jean Golding, Steven Gregory, Yasmin Iles-Caven, Joseph Hibbeln, Alan Emond, Caroline M Taylor

**Affiliations:** a Centre for Child and Adolescent Health, School of Social & Community Medicine, University of Bristol, Bristol, UK; b National Institute on Alcohol Abuse and Alcoholism, National Institutes of Health, Bethesda, MD, USA

**Keywords:** ALSPAC, Maternal blood mercury, Child Development, Selenium, Fish, Prenatal exposure

## Abstract

**Introduction:**

There is evidence that high levels of mercury exposure to the pregnant woman can result in damage to the brain of the developing fetus. However there is uncertainty as to whether lower levels of the metal have adverse effects on the development of the infant and whether components of fish consumption and/or the selenium status of the woman is protective.

**Methods:**

In this study we analysed data from the Avon Longitudinal Study of Parents and Children (ALSPAC) (*n* = 2875–3264) to determine whether levels of total blood mercury of pregnant women collected in the first half of pregnancy are associated with the development of the offspring at ages 6, 18, 30 and 42 months. The developmental measures used maternal self-reported scales for individual types of development (fine and gross motor, social and communication skills) and total scores. Multiple and logistic regression analyses treated the outcomes both as continuous and as suboptimal (the lowest 15th centile). The statistical analyses first examined the association of prenatal mercury exposure with these developmental endpoints and then adjusted each for a number of social and maternal lifestyle factors; finally this model was adjusted for the blood selenium level.

**Results:**

Total maternal prenatal blood mercury and selenium ranged from 0.17 to 12.76 and 17.0 to 324 μg/L respectively. We found no evidence to suggest that prenatal levels of maternal blood mercury were associated with adverse development of the child, even when the mother had consumed no fish during pregnancy. In general, the higher the mercury level the more advanced the development of the child within the range of exposure studied. For example, the fully adjusted effect sizes for total development at 6 and 42 months were +0.51 [95%CI +0.05, +1.00] and +0.43 [95%CI +0.08, +0.78] points per SD of mercury. For the risk of suboptimal development the ORs at these ages were 0.90 [95%CI 0.80, 1.02] and 0.88 [95%CI 0.77, 1.02]. In regard to the associations between blood mercury and child development there were no differences between the mothers who ate fish and those who did not, thus implying that the benefits were not solely due to the beneficial nutrients in fish.

**Conclusions:**

We found no evidence of adverse associations between maternal prenatal blood mercury and child development between 6 and 42 months of age. The significant associations that were present were all in the beneficial direction.

## Highlights

• Prenatal blood mercury was measured in >2000 women linked their offspring’s development at 4 time points.
• Adjusted associations indicated that the higher the level of blood mercury the more advanced the child’s development.
• Adjustment of the analyses for blood selenium did not alter the results.
• Separate analyses of women who consumed or did not consume fish produced similar results.
• There was no indication that child development is harmed by the levels of mercury we studied (up to the EPA’s RFD).

## 1 Introduction

In the Minamata tragedy during which a Japanese population consumed seafood (mainly shellfish) contaminated with very high levels of mercury, substantial brain damage resulted, especially to those individuals exposed in utero (Harada, 1968). Subsequently it has been assumed that even low levels of this toxic metal in pregnancy (which crosses the placenta easily) will have deleterious effects on the development of the brain of the offspring.

A number of studies have compared the maternal prenatal levels of mercury, either estimated from maternal hair, umbilical cord tissue or cord blood, with the cognitive development of the child. The results have been mixed, and varied with the type of seafood eaten in the area in which the studies were carried out. For example, in a review of the evidence Myers and Davidson (1998) noted that no adverse associations have been found in the Seychelles cohort study, where exposure to methyl mercury is mainly from fish consumption. In contrast, in the Faroe Islands where seafood exposure is primarily from consumption of whale meat and not fish, adverse associations have been reported (Grandjean et al., 1998); this difference may be because sea mammals are contaminated with multiple other toxicants.

In comparison with studies of school age children, there have been relatively few publications concerning preschool development in association with prenatal mercury exposure. Using the Bayley developmental scales a cohort of 374 children in Poland were examined at 12, 24 and 36 months but only the 12 month measure showed a significant deleterious outcome after adjustment (Jedrychowski et al., 2006, Jedrychowski et al., 2007). In the USA, Oken and colleagues reported that at 6 months there was a difference in novelty preference using a test of visual recognition memory (Oken et al., 2005), and at 3 years there were higher scores on tests of child development if the mother had eaten fish in pregnancy (Oken et al., 2008). The World Trade cohort used the Bayley MDI and PDI scales at ages 12, 24 and 36 months but only the 36 month measure showed a significant negative association of mercury with PDI, and only after controlling for fish intake (Lederman et al., 2008). These studies all involved relatively small numbers (<400), and tended to measure different end-points at different ages. They conveyed mixed messages concerning effects which may also have been the result of lack of power.

Not surprisingly there are also mixed messages from advisory bodies with somewhat arbitrary cut-offs for levels of concern of blood mercury levels for adults ranging from 2.0 μg/L in Germany to 5.8 μg/L in the USA (Taylor et al., 2014). However Raymond and Ralston (2004) postulated that “measuring the amount of mercury in the environment or food sources may provide an inadequate reflection of the potential for health risks if the protective effects of selenium are not also considered.”

We use a large prenatal birth cohort that includes measures of prenatal fish intake, blood selenium as well as blood mercury—the Avon Longitudinal Study of Parents and Children (ALSPAC). Adjusted analyses of this cohort have already shown that six of the 14 subcomponents of the total development scores showed significant trends with the amount of fish eaten, the less fish the mother ate, the more likely the child to be in the lowest quartile of ability (Hibbeln et al., 2007). This was particularly true of fine motor skills at 18 and 42 months, communication skills at 6 and 18 months and social skills at 30 and 42 months. No analyses of ALSPAC have yet determined whether the prenatal level of blood mercury is associated with these aspects of development, or with the total development scores.

The aims of this study therefore are to provide evidence to help elucidate:

(1) whether maternal prenatal blood mercury levels are associated with adverse preschool development;
(2) whether maternal blood selenium levels modify any associations with pre-school development;
(3) whether maternal fish consumption (with its beneficial levels of omega-3 fatty acids, vitamin D, choline and iodine (Wu et al., 2013) mask any adverse effects of maternal blood mercury on offspring development;
(4) whether the different components of the child development measures (fine and gross motor, communication and social skills) have differential associations.

## 2 Material and methods

### 2.1 The ALSPAC cohort

The ALSPAC study aimed to enrol all pregnant women residing in Avon (a geographically defined area that includes the city of Bristol, smaller urban towns, and rural areas about 120 miles west of London, UK) with an expected delivery date between 1 April 1991 and 31 December 1992. The study enrolled 14,541 pregnant women, estimated as about 80% of those eligible. Its stated aims were to evaluate genetic and environmental influences on health and development, including environmental factors measured prospectively during pregnancy (Golding et al., 2001, Boyd et al., 2013). Heavy metals were targeted in the planning for the study, which is why blood samples were obtained in acid-washed vacutainers (which was the advice at the time). In addition, detailed conversations at the planning stage with the late Dr David Horrobin resulted in the collection of dietary information to specifically identify possible effects of prenatal fatty acids (particularly oily fish) on the fetal brain. The study website contains details of all the data that are available through a fully searchable data dictionary: <http://www.bris.ac.uk/alspac/researchers/data-access/data-dictionary/http://www.bris.ac.uk/alspac/researchers/data-access/data-dictionary/>

### 2.2 Prenatal trace metal exposures

Blood samples specifically collected in acid-washed containers for determination of trace metals were obtained from 4484 women residing in two of the three Health Authority areas of the recruitment region. Samples were obtained by midwives as early as possible in pregnancy. The sociodemographic characteristics of the women who donated samples were comparable to those of the rest of the ALSPAC study population apart from including a slight excess of older and more educated mothers (Taylor et al., 2013). Gestational age at sample collection [known for 4472 mothers (99.7%)] had a median value of 11 weeks and mode of 10 weeks. The interquartile range (IQR) was 9–13 weeks, and 93% of the samples were collected at <18 weeks gestation. Samples were stored for 0–4 days at 4 °C at the collection site before being sent to the central Bristol laboratory. Samples were transported at room temperature for up to three hours, and stored at 4 °C as whole blood in the original collection tubes for 18–19.5 years before analysis.

Analyses of the blood samples were carried out by the Centers for Disease Control and Prevention (CDC) for whole blood mercury and selenium (CDC method 3009.1; unpublished information). Clotted whole blood was digested to remove all clots, before being analysed using inductively coupled plasma dynamic reaction cell mass spectrometry (ICP-DRC-MS) (PerkinElmer, 2001, Tanner and Baranov, 1999, Tanner et al., 2002, Thomas, 2003). The entire clotted whole blood was transferred to a digestion tube using concentrated nitric acid with the volume estimated from the weight (Taber, 1965). The blood sample was heated in a microwave oven at a controlled temperature and time during which the organic matrix of the blood was digested removing the clots. ICP-DRC-MS internal standards (iridium and tellurium) were added at a constant concentration to all blanks, calibrators, and samples (at the time of 1:9 dilution of digestate) to facilitate correction for instrument noise and drift. The standard additions method of calibration was used to optimize the analytical sensitivity of the method for the whole blood samples. A recovery spike was included in each analytical run for calibration verification and as a blind quality control (QC) sample. The ICP-DRC-MS was operated in the DRC mode using oxygen when analysing for mercury and selenium. QC materials as well as in-house QC samples with control limits unknown to the analysts were used for daily quality control. The limits of detection (LOD) were 0.24 μg/L for mercury; three samples were below this level and were ascribed a value of 0.7 times the LOD. There were no samples below the LOD for selenium.

We have shown elsewhere that the distribution of blood mercury levels in this pregnant population approximated to normal (Golding et al., 2013). The maternal blood mercury levels ranged from 0.17 to 12.76 μg/L, with 5th, 10th, 50th, 90th and 95th centiles of 0.81, 0.99, 1.86, 3.33 and 4.02 μg/L respectively. The concentrations were much higher for selenium, with range of 17.0 to 324.1 μg/L, and 5th, 10th, 50th, 90th and 95th centiles of 81.4, 86.7, 108.0, 139.0 and 152.5 μg/L.

### 2.3 Maternal dietary information

A questionnaire sent to the mother at 32 weeks gestation included a food frequency questionnaire (FFQ) comprising 103 food and drink items including three items related to seafood: white fish, oily fish, and shellfish. The participants were given guidelines to classify the three types using seafood categories that were most prevalent in the UK. Thus oily fish was described as including ‘pilchards, sardines, mackerel, tuna, herring, kippers, trout, salmon, etc.’; white fish as including ‘cod, haddock, plaice, fish fingers etc.’ and shellfish as including ‘prawns, crab, cockles, mussels, etc.’. The woman was asked how frequently she was currently eating each of these groups, with options: not at all; about once in 2 weeks; 1–3 times a week; 4–7 times a week; more than once a day’ (Rogers and Emmett, 1998).

### 2.4 Outcome measures

A battery of questions at ages 6, 18, 30 and 42 months was developed by ALSPAC from questions in the Denver Developmental Screening Test (Frankenburg and Dodds, 1967) resulting in scales specifically used by ALSPAC (Golding et al., 2016, In press). At 6 and 18 months this related to four different sub-categories: social skills, fine motor coordination, language and communication indicators and gross motor skills; the combined sub-categories provided a total development score. At 30 and 42 months, however, only three subgroups were used—these comprised social development, fine motor and gross motor development, and their combination provided a total development estimate. The basic numbers, ranges and medians of each score are shown in Table 1 and described in more detail elsewhere (Golding et al., 2016).

### 2.5 Potential confounders

We have shown elsewhere that maternal blood mercury is strongly related to a number of social factors that are irrespective of the maternal diet (Golding et al., 2013). We therefore allowed for these and other features known to be related to child development and/or mercury level; they comprise: a Family Adversity Index (Lereya and Wolke, 2013) derived from 38 factors relating to deprivation present in pregnancy including maternal depression and anxiety—used as a continuous scale; housing tenure (public housing v. rest); household crowding (no. of persons in household/no. of rooms available); level of maternal education; stressful life events in first half of pregnancy (sum of 44 possible events—treated as continuous scale); smoking at 18 weeks gestation (yes v. no); alcohol consumption at 18 weeks (yes v. no); maternal age at birth; parity (no. of previous deliveries); and whether the child was breast fed.

### 2.6 Statistical analyses

The statistical analyses first assessed the unadjusted associations between prenatal mercury and each of the developmental outcomes measured on continuous scales using multiple regression. Secondly, the analyses were adjusted for features of the child at the point at which he/she was assessed: term age at assessment (the length of time from the estimated time at which the child was due, thus taking account of the development of preterm infants) and sex; as well as all the confounders as described in 2.5 above (Model A). We did not adjust specifically for preterm delivery for two reasons: (a) only 1% of the infants were of gestation <33 weeks, and (b) we considered that if there was an adverse effect of mercury, this was likely to be on the causal pathway. Subsequently we incorporated selenium into the analyses by adding it as a continuous covariate (Model B). These models were then repeated for two subgroups: (a) for children whose mothers ate fish during pregnancy, and (b) those who did not.

A separate set of analyses was constructed to determine whether the lower 15% of the distribution (approximately 1SD below the mean) of the total development scores showed similar associations. We used logistic regression with this adverse outcome, and controlled for the same set of possible confounders as for the continuous data.

Since all the analyses were designed to determine whether any negative effects were apparent, and in order to avoid type 1 errors, we used *P* < 0.10 as our significance value.

## 3 Results

In Table 2 we illustrate the way in which the median and mean mercury levels vary with the potential confounders and the seafood variables. These highly significant associations demonstrate higher mercury levels related to increasing age of the mother, increasing educational achievements, increasing alcohol intake, and increasing seafood consumption. In contrast, mercury levels were lower among smokers, those living in rented accommodation and those who had had previous births (parity 1+).

Below we first examine the associations between prenatal mercury and the total development scores, since these outcomes are those most frequently used. Subsequently we describe the relationships with the subcomponents of the development score.

### 3.1 Relationships between blood mercury and mean total development scores

The ways in which the mean total development scores vary with increasing mercury intake are shown in Table 3. The left hand column shows that for three of the four ages the unadjusted relationships are positive—i.e., the higher the maternal mercury level the more able the young child. At 42 months, the association was statistically significant: *β* = +0.33, [95%CI + 0.03, +0.63]. When the children were divided according to whether or not their mothers had eaten fish during pregnancy, three-quarters of the associations were again positive.

Comparisons of the total development scores after adjustment using Model A are shown in Table 4. For all children combined (i.e., the left hand column), the effect sizes at each age were greater than for the unadjusted relationships in Table 3. All relationships were now positive and only the association at 30 months was not significant. For the children of women who ate fish, similar positive associations were shown for each stage of development using Model A, and for women who did not eat fish, the associations were also all positive; the effect sizes were often larger than those found among fish-eaters.

### 3.2 Relationships with risk of suboptimal development (<15th centile)

In Table 5 the risk of a child being in the lowest 15% of the total development score is given in terms of an odds ratio for each increasing standard deviation of mercury level. It can be seen that for all children combined and for each age, the unadjusted risk is below expectation (i.e., <1.00). This was true also for the children whose mothers had eaten fish, and for three of the four ages of assessment for the children whose mothers ate no fish.

Comparing the effect sizes after adjustment (Table 6), for all children and for those whose mothers ate fish, the odds ratios consistently remained less than 1.00 using Model A. For the children of the non-fish-eaters the confidence intervals of the odds ratios are wide, and therefore difficult to interpret—however, the adjusted odds of being less than 1SD in this group are consistently <1.00. This indicates that there is little likelihood of the child of the mother with high blood mercury being more likely to develop sub-optimally, although it cannot be ruled out.

### 3.3 Relationship between prenatal selenium levels and offspring development

Selenium was added to Model A to create Model B, and the regression coefficients are given in Table 4, Table 6 and in the accompanying paper (Golding et al., 2016). It can be seen from Table 4 that insertion of the selenium variable generally made little difference to the size of the positive associations between mercury and the developmental outcomes. Similarly adding selenium to the models made little difference to the odds ratios for suboptimal development (Table 6).

### 3.4 Relationship between maternal mercury levels and developmental subgroups

The results of adjusted analyses are shown for each developmental subgroup in Golding et al. (2016), and summarised in Table 7. Of the 14 fully adjusted outcomes tested using the complete sample, all were positively associated with prenatal exposure to mercury, eight of which were statistically significant at the 0.10 level; these involved social skills at 6, 18 and 42 months, fine motor coordination at 6, 18 and 42 months, and communication at 6 and 18 months. Separate analysis of the children born to women who were fish eaters again showed positive relationships between maternal mercury and the developmental scores for all variables, two being significant at the 0.05 level (social skills at 6 and 42 months). For the non-fish eaters there were negative associations for two of the measures (communication and gross motor at 6 months) but all others were positively associated with mercury level including three that were statistically significant at the 0.10 level (fine motor at 6 and 18 months and gross motor at 42 months).

## 4 Discussion

Although very high levels of prenatal mercury contamination have been shown to have deleterious effects on the intellectual development of the child, there is no evidence here to suggest that the levels found in this study (<12.8 μg/L) result in adverse development in the first years of the child’s life. On the contrary, in spite of detailed analysis using multiple confounders, we found positive (apparently beneficial) rather than negative associations. Adjustment for a variety of social and lifestyle factors tended to increase rather than decrease the size of the effects. Separate analysis of offspring born to women who ate fish in pregnancy showed similar positive beneficial associations, and analyses of women who were not fish eaters mostly tended also to have positive outcomes.

There has been much interest in the possible deleterious effects of prenatal exposure to mercury and possible preventive effects of selenium and/or seafood. In this study we have shown that the associations among fish eaters were similar in effect size to those among non-fish eaters, thus suggesting that consumption of fish was unlikely to account for the unexpected beneficial association. Similarly taking selenium into account in the analyses made little difference to the positive associations between level of mercury and child development, thus implying that selenium was not masking any deleterious effects of mercury.

We have also tested the data to determine whether there might be an adverse effect in the lower tail of the developmental distribution that might be masked by looking at the overall mean developmental levels, but we found that even on adjustment the higher the mercury level the lower the risk of the child being in the lower (suboptimal) tail. Again, our analyses taking selenium into account, or assessing the relationships within the fish and non-fish consumers made little difference to these conclusions.

A major reason for looking separately at the offspring of the fish eaters concerns the fact that, in general, these mothers have higher levels of mercury (since fish is a major dietary source of mercury), but also have increased levels of nutrients known to be beneficial to the developing fetus such as iodine, vitamin D, choline and essential fatty acids such as the omega-3s (Wu et al., 2013). Therefore it could be that this group may have a different (less toxic) relationship between mercury and development than that found among offspring of women who ate no fish. In the event we found no demonstrable difference between the effect sizes of the two groups, and in some instances an enhanced positive association in the non-fish eaters in regard to overall development at 42 months (Table 4), and gross motor coordination at 42 months (Table 7).

This is in contrast with the results found when assessing the associations with the child’s IQ measured later in childhood. Using the same strategy as in this paper, we found similar beneficial associations between mercury level and IQ if the mother consumed fish, but if she ate no fish, there were negative associations. The negative associations were particularly apparent for performance IQ rather than verbal IQ. Consequently in this study we looked especially at the less verbal developmental outcomes, expecting that there might be evidence of negative associations among the fine motor and the gross motor developmental scores. We found no such associations.

There are a number of strengths and limitations to this project. The strengths are: (a) It is based on a geographic population with a high enrolment rate (∼80%), and consequently may be more generalisable. (b) The numbers involved in the analyses were substantially larger than reported in other studies, and consequently are statistically more powerful. (c) A relatively large number of confounders were available to be taken into account, thus diminishing likelihood of bias in the results. (d) The prenatal data were collected prospectively with no knowledge as to how well the child would develop, again reducing the likelihood of possible bias.

In contrast the limitations are: (i) The child’s developmental scores were obtained from maternal report rather than examination by a trained assessor—although there was evidence of good concordance between these scores and examination by trained psychologists (Golding et al., 2016). (ii) Clearly in analyses of the effects of prenatal measures on the offspring, the sources of mercury in the population may be important confounders. We have shown elsewhere that in the ALSPAC population, the diet contributes about 20% of the variance of prenatal whole blood mercury, almost half of this being contributed by seafood (Golding et al., 2013). We have also shown that the amount of dental amalgam in the woman’s mouth during pregnancy contributes a further 6.5% of the variance (Golding et al., 2015)]. The latter study showed that once diet and dental factors had been taken into account, the social factors that were still associated with the prenatal mercury levels were maternal age, parity, and maternal education. We have therefore taken these social and biological factors into account, together with a number of others associated with child development, in our analyses. Although we may not have allowed for key variables in the regression analyses, we found that taking account of the above variables mostly resulted in an increase, rather than a decrease in the positive effect sizes. Thus allowing for further social variables is unlikely to reveal a negative association with child development. (iii) The measures of mercury and selenium were obtained from whole blood in the first half of pregnancy—it may be that exposure later in pregnancy may be more deleterious. However, also using ALSPAC data, Daniels et al. (2004) showed a similar lack of association with child development at 18 months using mercury measured in the umbilical cord tissue, reflecting exposure in late pregnancy. (iv) The levels of total blood mercury in this population may not be relevant to other populations and therefore our results may not be generalisable. However review of ALSPAC cord tissue levels by the FDA concluded that the levels were very similar to those found in NHANES, and that they were likely to be comparable with the USA in general (http://www.fda.gov/Food/FoodborneIllnessContaminants/Metals/ucm173408.htm accessed 30.01.16.). The selenium levels were likewise not excessive with a 99th percentile at 194.9 μg/L. The 99th percentile for the molar mercury-selenium ratio was 0.02 (data not shown), which is well below the level (>1) where mercury toxicity has been observed to occur (data not shown).

## 5 Conclusions

Our results show beneficial associations between maternal blood mercury levels in the range we studied and early child development. These beneficial associations were strengthened by adjustment for a variety of covariates. Since we studied levels of prenatal mercury exposure (median 1.86 μg/L) present in most Western countries, these results are likely to have direct relevance to other developed nations.

## Competing financial interests

The authors have no competing interests.

## Figures and Tables

**Table 1:** Summary of basic statistics relating to child development scores using questionnaire measures.

Age	Score	No. tested	Range	Median	Mean [SD]
6 m	Social skills	11354	0–30	17	17.23 [4.86]
Fine motor	11359	0–33	21	21.13 [6.36]
Communication	11355	0–24	16	16.44 [2.97]
Gross motor	11394	0–39	16	16.42 [5.93]
Total	11348	6–126	70	71.23 [15.57]

18 m	Social skills	11087	0–28	19	19.15 [3.89]
Fine motor	11090	0–32	27	26.69 [3.06]
Communication	11102	0–28	16	15.94 [4.79]
Gross motor	11087	0–22	20	19.45 [2.88]
Total	11067	4–110	82	81.25 [10.78]

30 m	Social skills	10260	0–26	19	18.94 [3.76]
Fine motor	10286	0–32	27	26.37 [3.76]
Gross motor	10272	0–22	20	19.51 [2.52]
Total	10244	0–80	66	64.87 [7.82]
					
42 m	Social skills	10014	0–26	23	22.10 [3.14]
Fine motor	10019	0–34	31	29.72 [4.06]
Gross motor	10026	0–30	27	26.35 [3.59]
Total	10011	0–90	80	78.19 [8.83]

**Table 2:** Mean and median levels of prenatal blood mercury associated with maternal features.

	*N*	Median	Mean (SD)	*R*^2^ (%)
Maternal age				
<20	239	1.34	1.50 (0.74)	
20–24	813	1.57	1.79 (1.00)	
25–29	1531	1.90	2.11 (1.11)	
30–34	1019	2.12	2.29 (1.07)	
35+	311	2.18	2.44 (1.21)	4.87

Parity				
0	1622	1.97	2.22 (1.24)	
1	1249	1.86	2.02 (0.95)	
2	540	1.86	2.02 (0.90)	
3+	239	1.68	1.86 (0.95)	0.96

Maternal education				
A (lowest)	673	1.54	1.75 (0.95)	
B	335	1.73	1.89 (1.01)	
C	1155	1.88	2.03 (1.03)	
D	802	2.05	2.29 (1.16)	
E (highest)	547	2.40	2.60 (1.18)	5.98

Smoked mid-pregnancy				
Yes	752	1.61	1.83(0.97)	
No	2968	1.96	2.16(1.11)	1.50

Alcohol consumption (units) mid pregnancy				
Not at all	1789	1.76	1.96(1.05)	
<1/week	1228	1.95	2.17(1.07)	
1–6/week	550	2.09	2.28(1.06)	
1+/day	65	1.94	2.39(1.56)	1.44

Housing tenure				
Owned/mortgaged	2695	2.00	2.20(1.10)	
Council rented (public housing)	570	1.52	1.71(0.96)	
Other	444	1.79	2.02(1.18)	2.61

Frequency mother ate white fish				
Never/rarely	644	1.39	1.63 (1.02)	
Once in 2 weeks	1396	1.92	2.09 (0.99)	
1–3 times/week	1374	2.10	2.35 (1.14)	
4+ times/week	49	2.08	2.34 (1.15)	5.15

Frequency mother ate oily fish				
Never/rarely	1479	1.55	1.75 (0.94)	
Once in 2 weeks	1139	2.08	2.28 (1.08)	
1–3 times/week	803	2.27	2.52 (1.16)	
4+ times/week	42	2.36	2.38 (1.02)	8.13

Frequency mother ate shellfish				
Never/rarely	2766	1.83	2.02 (1.05)	
Once in 2 weeks	565	2.25	2.48 (1.13)	
1+ times/week	132	2.28	2.53 (1.30)	2.74

Each continuous variable is statistically significant at *P* for trend <0.0001.

**Table 3:** Unadjusted relationship (change in development score for each SD of mercury) between prenatal mercury exposure and the child’s score on the total development scale. Highlighted are results with *P* < 0.100.

Age at measurement	All children		Mother ate fish		Mother ate no fish	
	*N*	*β* [95% CI]	*N*	*β* [95% CI]	*N*	*β* [95% CI]
6 m	3264	+0.34 [−0.10,+0.77]	2648	**+0.52 [+0.05,+0.99]**	435	+0.04 [−1.73,+1.81]
		(*P* = 0.131)		**(*****P*** **=** **0.031)**		*P* = (0.962)
18 m	3162	+0.22 [−0.12,+0.56]	2596	+0.16 [−0.22,+0.53]	404	+0.23 [−1.00,+1.47]
		(*P* = 0.200)		(*P* = 0.411)		(*P* = 0.710)
30 m	3009	−0.04 [−0.30,+0.22]	2472	−0.07 [−0.35,+0.22]	386	−0.09 [−1.04,+0.86]
		(*P* = 0.748)		(*P* = 0.649)		(*P* = 0.849)
42 m	2875	**+0.33 [+0.03,+0.63]**	2363	+0.24 [−0.09,+0.57]	372	+0.69 [−0.55,+1.92]
		(***P*** **=** **0.032)**		(*P* = 0.156]		(*P* = 0.274)

+ve sign indicates a beneficial association with development; −ve sign indicates the opposite.

**Table 4:** Relationship (change in points of development score for each SD of mercury) between prenatal mercury exposure and child development score after adjustment for A (age at assessment and sex of child, maternal age, parity, education, smoking, alcohol, housing tenure, household crowding, family adversity score, life events in the first half of pregnancy and whether the child was breast fed), and B (A + prenatal selenium). Highlighted are results with *P* < 0.100.

Age at measurement	All children		Mother ate fish		Mother ate no fish	
	*N*	*β* [95% CI]	*N*	*β* [95% CI]	*N*	*β* [95% CI]
6 m						
Model A	2721	**+0.55 [+0.10,+1.00]**	2354	**+0.60 [+0.13,+1.08]**	354	+0.25 [−1.67,+2.17]
		(***P*** **=** **0.017)**		**(*****P*** **=** **0.012)**		(*P* = 0.796)
Model B	2721	**+0.51 [+0.05,+1.00]**	2354	**+0.55 [+0.06,+1.04]**	354	+0.28 [−1.65,+2.21]
		(***P*** **=** **0.031)**		**(*****P*** **=** **0.027)**		(*P* = 0.777)
18 m						
Model A	2643	**+0.47 [+0.09,+0.84]**	2294	**+0.35 [−0.05,+0.75]**	337	+0.96 [−0.43,+2.36]
		(***P*** **=** **0.015)**		**(*****P*** **=** **0.086)**		(*P* = 0.175)
Model B	2643	**+0.49 [+0.10,+0.88]**	2294	**+0.40 [−0.02,+0.81]**	331	+0.94 [−0.46,+2.34]
		(***P*** **=** **0.013)**		**(*****P*** **=** **0.059)**		(*P* = 0.186)
30 m						
Model A	2452	+0.15 [-0.15,+0.44]	2124	+0.08 [−0.24,+0.39]	317	+0.55 [−0.47,+1.57]
		(*P* = 0.331)		(*P* = 0.628)		(*P* = 0.290)
Model B	2452	+0.23 [−0.08,+0.53]	2124	+0.18 [−0.15,+0.51]	317	+0.55 [−0.48,+1.57]
		(*P* = 0.148)		(*P* = 0.291)		(*P* = 0.297)
42 m						
Model A	2394	**+0.38 [+0.04,+0.72]**	2073	+0.26 [−0.10,+0.62]	317	**+1.16 [−0.21,+2.54]**
		(***P*** **=** **0.030)**		(*P* = 0.160)		**(*****P*** **=** **0.096)**
Model B	2394	**+0.43 [+0.08,+0.78]**	2073	+0.31 [−0.07,+0.68]	317	**+1.26 [−0.12,+2.64]**
		(***P*** **=** **0.017)**		(*P* = 0.110)		**(*****P*** **=** **0.074)**

+ve sign indicates a beneficial association with development; −ve sign indicates the opposite.

**Table 5:** The unadjusted odds of the child scoring <1 SD (<15% percentile) of the total development score in relation to each SD of maternal prenatal mercury level. Highlighted are results with *P* < 0.100.

Age at measurement	All children		Mother ate fish		Mother ate no fish	
	*N*	OR [95% CI]	*N*	OR [95% CI]	*N*	OR [95% CI]
6 m	3264	**0.90 [0.82,0.99]**	2648	**0.88 [0.79,0.98]**	435	0.90 [0.63,1.28]
		(***P*** **=** **0.028)**		**(*****P*** **=** **0.022)**		(*P* = 0.541)
18 m	3162	**0.89 [0.80,0.98]**	2596	**0.89 [0.80,1.00]**	404	0.96 [0.69,1.33]
		(***P*** **=** **0.023)**		**(*****P*** **=** **0.052)**		(*P* = 0.802)
30 m	3009	0.96 [0.87,1.05]	2472	0.96 [0.87,1.06]	386	1.08 [0.82,1.42]
		(*P* = 0.342)		(*P* = 0.408)		(*P* = 0.597)
42 m	2875	**0.88 [0.79, 0.97]**	2363	**0.89 [0.79,1.00]**	372	0.84 [0.58,1.23]
		(***P*** **=** **0.013)**		**(*****P*** **=** **0.048)**		(*P* = 0.376)

An OR <1.00 indicates a lower risk of subnormal development with each SD of mercury level.

**Table 6:** The odds of the child scoring <1 SD (15th centile) on the child development scale in relation to each SD of maternal prenatal blood mercury after adjustment for: A (age at assessment and sex of child, maternal age, parity, education, smoking, alcohol, housing tenure, household crowding, family adversity score, life events in the first half of pregnancy and whether the child was breast fed), and B (A + prenatal selenium). Highlighted are results with *P* < 0.100.

Age at measurement	All children		Mother ate fish		Mother ate no fish	
	*N*	OR [95% CI]	*N*	OR [95% CI]	*N*	OR [95% CI]
6 m						
Model A	2721	**0.89 [0.79, 1.01]**	2354	**0.89 [0.78, 1.01]**	354	0.98 [0.64, 1.51]
		(***P*** **=** **0.060)**		**(*****P*** **=** **0.073)**		(*P* = 0.935)
Model B	2721	**0.90 [0.80,1.02]**	2354	0.90 [0.79,1.03]	354	0.98 [0.64,1.51]
		(***P*** **=** **0.099)**		(*P* = 0.127)		(*P* = 0.932)
18 m						
Model A	2643	**0.86 [0.76,0.97]**	2294	**0.87 [0.76,0.99]**	337	0.86 [0.59,1.26]
		(***P*** **=** **0.016)**		**(*****P*** **=** **0.038)**		(*P* = 0.438)
Model B	2643	**0.85 [0.75,0.97]**	2294	**0.85 [0.74,0.98]**	337	0.87 [0.59,1.26]
		(***P*** **=** **0.015)**		**(*****P*** **=** **0.026)**		(*P* = 0.453)
30 m						
Model A	2452	**0.90 [0.80,1.01]**	2124	0.93 [0.82,1.06]	317	0.78[0.53,1.15]
		(***P*** **=** **0.068)**		(*P *= 0.269)		(*P* = 0.213)
Model B	2452	**0.90 [0.80,1.01]**	2124	0.92[0.81,1.05]	317	0.79 [0.54,1.17]
		(***P*** **=** **0.072)**		(*P* = 0.221)		(*P* = 0.244)
42 m						
Model A	2394	**0.89 [0.78,1.01]**	2073	0.92 [0.81, 1.05]	311	0.73 [0.45,1.18]
		(***P*** **=** **0.063)**		(*P* = 0.240)		(*P* = 0.193)
Model B	2394	**0.88 [0.77,1.00]**	2073	0.92 [0.80,1.05]	311	0.71 [0.44,1.16]
		(***P*** **=** **0.052)**		(*P* = 0.210)		(*P* = 0.193)

An OR <1.00 indicates a lower risk of subnormal development with each SD of mercury level.

**Table 7:** The significance of the results of the adjusted analyses of each of the sub-categories of the development scores for each of the 4 ages as depicted in Golding et al. (2016). The results are shown as follows: 0 = *P* ≥ 0.10; + = *P* < 0.10; ++ = *P* < 0.05; +++ *P *< 0.01.

Developmental level	All children	Mothers ate fish prenatally	Mothers did not eat fish
Social skills			
- 6 m	+++	+++	0
- 18 m	+	0	0
- 30 m	0	0	0
- 42 m	+++	++	0
Fine motor			
- 6 m	+	0	+
- 18 m	++	+	++
- 30 m	0	0	0
- 42 m	+	0	0
Communication			
- 6 m	+	0	0
- 18 m	+	0	0
Gross motor			
- 6 m	0	0	0
- 18 m	0	0	0
- 30 m	0	0	0
- 42 m	0	0	+++

+Indicate positive associations; there were no negative associations.
